# A case report on a protracted course of a hidden insulinoma

**DOI:** 10.1016/j.amsu.2021.102240

**Published:** 2021-03-24

**Authors:** Fatimah Zaherah Mohamed Shah, Aimi Fadilah Mohamad, Nur Aisyah Zainordin, Nur’ Aini Eddy Warman, Sharifah Faradila Wan Muhamad Hatta, Rohana Abdul Ghani

**Affiliations:** a Endocrine Unit, Department of Internal Medicine, Faculty of Medicine, Universiti Teknologi MARA (UiTM), 47000, Jalan Hospital, Sungai Buloh, Selangor, Malaysia; b Hospital Sg Buloh, 47000, Jalan Hospital, Sungai Buloh, Selangor, Malaysia; c Institute of Pathology, Laboratory and Forensic Medicine (I-PPerForM), Sg Buloh Campus, Universiti Teknologi MARA (UiTM), 47000, Jalan Hospital, Sungai Buloh, Selangor, Malaysia

**Keywords:** Insulinoma, Hypoglycemia, Neuroendocrine tumor, Endogenous hyperinsulinemia, Selective arterial calcium stimulation test (SACST), Case report, Selective Arterial Calcium Stimulation Test (SACST), Pancreatic Neuroendocrine Tumors (p-NET)

## Abstract

**Introduction:**

Insulinoma is a functioning pancreatic neuroendocrine tumor primarily leading due to hypoglycemia due to hypersecretion of insulin. This case illustrates the real challenges faced in the detection of an occult insulinoma, which resulted in a protracted course of the disease.

**Case presentation:**

A 33-year-old female presented with recurrent hypoglycemia. Endogenous hyperinsulinemia was confirmed by a prolonged fast, however serial imaging was negative. Incidental finding of an ovarian mass gave rise to the suspicion of an insulin-producing ovarian tumor. Subsequent multimodality pancreatic imaging remained negative, requiring more invasive investigations. The tumor was localized by specialized arteriography using calcium stimulation to support the diagnosis of an insulinoma. However, repeated negative imaging led to further delays in definitive management, with worsening hypoglycemia. The surgery was finally performed three years after the initial presentation with successful removal of the tumor using intra-operative ultrasound.

**Clinical discussion:**

It is important to emphasize that preoperative radiological imaging is useful to localize pancreatic lesions. However, most insulinomas could only be detected intraoperatively. The absence of suggestive radiological evidence should not deter surgeons from proceeding with definitive surgical intervention.

**Conclusion:**

The case highlights the importance of a multidisciplinary approach in the management of a complicated case.

## Highlights

• Insulinoma is a difficult disease to diagnose due to its ambiguous location and insidious course.
• Insulinoma and type 2 diabetes mellitus coexistence is rare.
• Radiological imaging has improved in aiding the detection of pancreatic lesion but falsely negative results remain high.
• Surgical intervention is imperative for explorative diagnosis as well as treatment.
• A multidisciplinary approach could have been more successful in early detection and thus providing definitive treatment.

## 1 Introduction

Insulinoma is a rare neuroendocrine tumor with an annual incidence of 1–3 per million population per year [1]. While unprovoked hypoglycemia is a well-known presentation, the diagnosis is often delayed due to difficulties in identifying and localizing the functioning tumor. We report a case of an unfortunate young lady who suffered a prolonged course of the disease before finally receiving a definitive and curative treatment. The work has been reported in line with the SCARE criteria [2].

## 2 Case

A 33-year-old woman initially presented with osmotic symptoms and a fasting blood glucose of 9 mmol/L, and was started on metformin and low dose gliclazide. Episodes of hypoglycemia started 3 months later, prompting the cessation of both these medications. However, her symptoms persisted and she experienced episodes of palpitation, sweating and dizziness at her workplace. Upon arrival at the Emergency Room, her blood glucose was confirmed to be below 3 mmol/L, and her symptoms improved following dextrose infusion. Physical examination revealed a well-built woman with a body mass index of 28.4kg/m2 and other examinations were unremarkable. She gave further history of intermittent dizziness, palpitation and sweating during exercise and while performing house chores within the last few months in the absence of any anti-diabetic medications. These symptoms occurred at any time of the day, with no clear predilection for the fasting periods. She had also been experiencing gradual weight gain over the last few months, but denied changes in behavior or morning headaches, and has not been taking traditional medications or supplements. She had no previous medical, surgical nor psychological history, and she had not been taking any prescribed medication. Interestingly, her late father who had hypertension and end-stage renal disease on dialysis was diagnosed with insulinoma 2 years previously, but unfortunately succumbed to septicemic shock secondary to pneumonia. Her 8 siblings had been well with no known medical illness. She had no allergies and never smoked. She lived with her sister and was financially independent as a shop assistant.

She was admitted and further investigated for unprovoked hypoglycemia. Her baseline blood results, including her hemoglobin, white cell count, platelets, renal and liver profiles were all within normal ranges. She was commenced on a prolonged fast, and developed hypoglycemia within 9 hours of fasting, with symptoms of sweating, palpitations and drowsiness, confirmed by a capillary blood glucose of 2.8mmol/l.

The results were suggestive of insulinoma, with venous blood glucose of 2.3 mmol/l, Insulin 24 uIU/L (6-27) and C-Peptide 1685 pmol/L (165-662). However, subsequent computed tomography (CT) scan with pancreatic protocol did not reveal any significant pancreatic lesions. In view of the family history of insulinoma, the possibility of Multiple Endocrine Neoplasia (MEN) 1 was entertained. However, further investigations revealed negative results (Table 1). She was planned for further diagnostic workup, including an endoscopic ultrasound, unfortunately she was lost to subsequent follow-up for the next few months.

**Table 1:** Hormonal profile for the possibility of Multiple Endocrine Neoplasia (MEN) 1.

Hormonal Profile	Results	Normal Range
Thyroid Stmulating Hormone (mIU/L)	1.089	0.5–4.67
Free thyroxine (pmol/L)	13.95	9.14–23.81
Adrenocorticotrophic Hormone (ACTH) (pmol/L)	1.9	<11
Cortisol (early morning) (nmol/L)	348	250–500
Follicle Stimulating Hormone (FSH) (IU/L)	5.62	0.95–11.95
Luteinising Hormone (LH) (IU/L)	4.92	1.3–12.9
Estradiol (pg/mL)	20	8.4–28.7
Prolactin (µIU/mL)	264	59–169

**Fig. 1:**
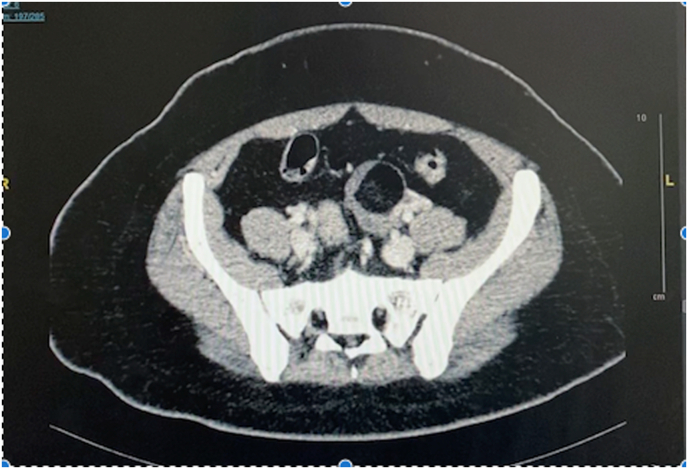
Computed tomography (CT) scan of the abdomen showing a left ovarian mass measuring 3.6 × 3.7 × 4cm, with features suggestive of a teratoma.

She presented six months later to a gynecologist with lower abdominal pain and irregular menses. She had learnt to overcome the intermittent symptoms of hypoglycemia in the interim by eating regular small meals. A computed tomography scan of the abdomen showed no pancreatic lesions, but revealed a left ovarian mass measuring 3.6 × 3.7 × 4cm, with features suggestive of a teratoma. Refer Fig. 1. The possibility of an insulin-producing ovarian teratoma was therefore considered, and while waiting for surgery, continuous unprovoked hypoglycemia necessitated diazoxide therapy, with some alleviation in her symptoms. She subsequently underwent laparoscopic left oopherectomy a few weeks later, and the histopathological examination reported a benign dermoid cyst. Unfortunately, the hypoglycemic episodes persisted after surgery requiring higher doses of diazoxide.

**Fig. 2:**
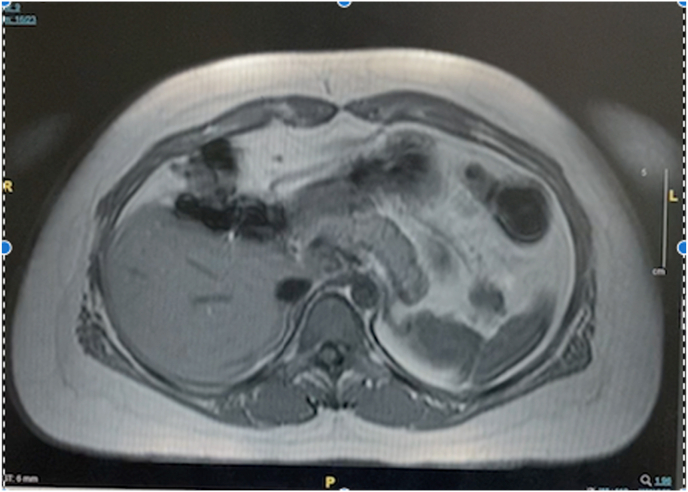
Magnetic Resonance Imaging (MRI) of the pancreas showing no obvious lesion in the pancreas.

She underwent another battery of investigations for localization of the insulin-producing lesion, including a repeated CT pancreatic protocol, endoscopic ultrasound and magnetic resonance imaging (MRI) of the pancreas, all of which failed to reveal any significant abnormalities. Refer Fig. 2. She subsequently underwent a selective arterial calcium stimulation test. This is an arteriography requiring catheterization of arterial branches of the celiac system supplying the pancreas, with calcium injections and simultaneous measurement of the hepatic vein insulin. An increase in insulin level accompanied by a raised c-peptide localizes the source of endogenous hyperinsulinism to a specific part of the pancreas, namely the pancreatic head, body or tail.

The results of the SACST (Table 2) was highly suggestive of localization towards the region supplied by the splenic artery, corresponding to the tail of pancreas. However, as the various imaging results were unable to confirm a definitive lesion, surgery was deemed inappropriate at that time. She continued to have intermittent episodes of hypoglycemia requiring increasing doses of diazoxide, and even short courses of prednisolone during hospital admissions for severe hypoglycemia. These caused significant disruptions in her work schedule, which led to termination of her employment, and subsequent financial and psychological burden. We continued to monitor her condition with periodic imaging of the pancreas and intermittent discussions with the surgeon. Due to the gradual worsening of her symptoms, a distal pancreatectomy was finally performed by a group of Endocrine and Hepatobiliary surgeons, almost 3 years after her initial presentation. Intraoperative ultrasonography showed a heterogenous hyperechoic lesion measuring 10.1 mm × 8.2 mm at the tail of pancreas, which corresponded to the thickened area palpated at the tail of pancreas. Histopathological examinations confirmed an islet cell hyperplasia. Her symptoms resolved almost immediately following the surgery. Approximately 4 months later she demonstrated levels of hyperglycemia, and was started on oral anti-diabetic medications. Her quality of life improved considerably, she was able to comply to the medications prescribed and was able to return to work. The patient remained under our follow up with regular biochemical monitoring. From her perspective, she initially could not understand the delay in the diagnosis and the need for the series of tests that she had to endure. Nevertheless, she remained positive throughout the whole challenging experience. She expressed her utmost appreciation and gratitude to the teams of physicians and surgeons involved in her management. She provided written informed consent for the publication of this case report and its related images.

**Table 2:** Results of the selective arterial calcium stimulation test (SACST).

Table 2a. SACST - Insulin levels demonstrating localization towards the region supplied by the splenic artery, corresponding to the tail of pancreas			
Insulin (uIU/ml)	Gastroduodenal Artery	Superior Mesenteric Artery	Splenic Artery
0 sec	33.7	27.9	32.0
30 sec	42.7	27.4	**89.0**
60 sec	39.5	24.6	**102.0**
120 sec	34.9	28.3	**63.8**

Table 2b. C-Peptide levels confirms localization towards the region supplied by the splenic artery, corresponding to the tail of pancreas			
C-Peptide (pmol/L)	Gastroduodenal Artery	Superior Mesenteric Artery	Splenic Artery
0 sec	516	312	536
30 sec	867	331	**2969**
60 sec	636	300	**2082**
120 sec	573	316	**1003**

## 3 Discussion

Insulinomas are rare with an estimated incidence of 1–3 per million population per year [1]. It however remains as one of the most common functioning neuroendocrine tumors of the pancreas (25% of endocrine pancreatic tumors), and also the commonest cause of hyperinsulinemic hypoglycemia [1,3]. The vast majority of cases (90%) are sporadic with solitary tumors, whereas approximately 5–10% are multiple and associated with multiple endocrine neoplasia (MEN) 1. Incidence of insulinoma peaks in the fifth decade, with a slight female preponderance (female to male ratio 1.4:1), whereas MEN 1 patients present at a younger age [3,4].

Unprovoked hypoglycemia is the pathognomonic feature of insulinoma, with an episodic nature, which reflects the intermittent secretion of insulin by the tumor. The main symptoms are either autonomic symptoms including palpitation, irritability and sweating, or due to the effects of hypoglycemia on the central nervous system, such as confusion, headache and behavioral changes [1,3]. Although fasting hypoglycemia has long been recognized as the main presentation of insulinoma, but it has now been increasingly acknowledged that patients may also present with postprandial hypoglycemic symptoms, and the most common cause of endogenous hyperinsulinism is insulinoma [1]. Hence, the investigation for insulinoma commenced for the case illustrated above.

In endogenous hyperinsulinism, the cause for hypoglycemia is the low levels of glucose production rather than increased utilization of glucose. Hence the main point is the failure of suppression of insulin secretion during this low glucose level [3]. This lends credence to the fact that plasma insulin, C-peptide and proinsulin levels may not be elevated relative to normal euglycemic values, but rather, inappropriately high when fasting plasma glucose concentration is low. The gold standard for biochemical detection of hypoglycemia disorders, especially insulinoma, remains the prolonged fast, with measurements of plasma insulin, C-peptide and proinsulin during hypoglycemia. This test can detect almost 99% of insulinomas [4,5]. Although the optimal duration has been debated, and traditionally taken as a 72-h fast, patients with insulinoma generally tend to develop hypoglycemia much sooner [5]. This was in keeping with our patient, who developed clinical and biochemical hypoglycemia within 9 hours of fasting, with inappropriately high insulin and C-peptide concentrations during the episode. The highly suggestive initial result underscored the importance in pursuing the diagnosis of insulinoma in this patient.

Insulinomas are almost universally located within the pancreas, generally described as 1/3 in the head, body and tail of the pancreas [3], thus concentrating the localization mainly within the pancreas. However, there have been reports of ectopic insulin secreting tumors, particularly neuroendocrine tumors in the abdomen and pelvis [6,7]. Of special interest to this case is the description of insulin producing ovarian tumors, with a mini-review of 7 cases highlighting the predominance of ovarian carcinoid giving rise to this phenomenon [8]. In our patient illustrated above the initial and subsequent negative imaging of the pancreas, coupled with the finding an ovarian tumor suggestive of a teratoma, resulted in the diagnosis of an insulin secreting ovarian tumor be entertained. Because of the persistently debilitating and undoubtedly worsening symptoms of hypoglycemia, she underwent removal of the tumor. Unfortunately, the ovarian mass proved to be a red herring, as she continued to have similar symptoms post-operatively.

Localization of the insulin-producing tumor was indeed the most challenging part in this patient's journey towards a definitive cure. She underwent an extensive series of imaging modalities with no amenable positive result. Conventional studies namely transabdominal ultrasonography, computed tomography and magnetic resonance imaging have varying detection rates, where some series are positive in only 10-40% of cases, versus a sensitivity approaching 70-85% for CT and MRI in other reports [1,3,4]. This underscores the fact that a negative imaging does not exclude insulinoma, particularly as insulinomas are often less than 1cm in diameter. Endoscopic pancreatic ultrasonography is an invasive technique, with positivity of 70–95% of cases in centers with experienced endoscopists and is the study of choice if the non-invasive imaging modalities yield negative results [1,3]. However, it is limited by its poor evaluation of lesions at the distal body or pancreatic tail with detection rate of 37–60% in comparison to 83-100% of lesions in the head and body of the pancreas [4], which could have explained the initial negative result in our patient. Newer non-invasive imaging modalities unfortunately have limited sensitivity. Somatostatin receptor scintigraphy is reported to detect only 50% of localized insulinomas because of either low density or lack of somatostatin receptors with highly avid affinity for octreotide. The relative utility of positron emission tomography (PET) has yet to be determined [1,3].

The subsequent protracted course of insulinoma in this case further highlights the challenges in localization of cases with negative imaging. After the negative imaging results, she underwent another invasive modality, namely selective arterial calcium stimulation test (SACST) for localization of this elusive tumor. Similar to other pancreatic neuroendocrine tumors (p-NET), insulinoma is a vascular tumor. Hence, selective angiography has been shown to be positive in 60% of cases and when combined with hepatic venous sampling after intra-arterial calcium administration it regionalizes insulinoma with high sensitivity, with reported positive detection rates of 88–100% [3,4]. The SACST technique utilizes the release of insulin from abnormal pancreatic β-cells but not normal β-cells the following exogenous intra-arterial calcium stimulation. A positive test requires a minimal 2-fold rise in hepatic insulin level from baseline [9,10]. It is important to highlight here that the test is essentially a preoperative investigation to assist in localization of the tumor. In reference to this case, although the SACST examination was subsequently able to localize the insulin-secreting lesion to the tail of pancreas, surgical intervention was delayed due to lack of conclusive radiological evidence. Thus, the SACST eventually served as a diagnostic test to prompt a definitive surgical intervention.

Interestingly, although some authors insist on preoperative localization with SACST, others have suggested that it may not be essential as most insulinomas can be detected intraoperatively [4,11]. In a review by Ravi et al. of 20 patients with sporadic insulinomas, of which 5 were occult, it was reported that intra-operative inspection and palpation localized the lesions in 91% of cases, whilst intra-operative ultrasound (IOUS) was able to do the same in 93% of cases. Combining these two methods resulted in almost 100% sensitivity. In addition, the site and size of the tumors were reported to correlate poorly with pre-operative localization [11]. It is therefore unfortunate that this patient had to endure repeated examinations, suffered through continuous episodes of hypoglycemia that significantly affected her quality of life and imposed financial burden that required the assistance from the state. She finally underwent the imperative and curative exploratory surgery, assisted by intraoperative ultrasound that effectively localized the lesion and subsequently resulted in a successful distal pancreatectomy. Post operatively her quality of life improved significantly. She was able to resume her employment with minimal number of sick days. She was married a year after the surgery and to date she is well and happy in her endeavor towards a complete family.

## 4 Conclusion

Although rare, insulinomas remain as one of the commonest neuroendocrine tumors of the pancreas, with episodes of debilitating hypoglycemia and reduced quality of life, requiring early detection and intervention. The sensitivity of a variety of imaging examinations are variable, with SACST having the highest success rate. Surgery is a definitive treatment but frequently delayed because of limited radiographic evidence. This case, therefore, clearly illustrates some of the challenges posed in the detection and management of occult insulinoma. It certainly underscores the importance of a multidisciplinary approach and cooperation from all members to be well informed in the most recent scientific knowledge in managing an extremely complicated and intriguing case.

## Consent

Written informed consent was obtained from the patient for publication of this case report and accompanying images. A copy of the written consent is available for review by the Editor-in-Chief of this journal on request.

## Ethical approval

None declared.

## Sources of funding

Partial reimbursement of publication fee from 10.13039/501100004625Universiti Teknologi MARA.

## Author contribution

All authors provided clinical input to the case report and comments towards the manuscript.

## Trial registry number

None.

## Guarantor

Prof Dr Rohana Abdul Ghani, Consultant Endocrinologist, UiTM.

## Provenance and peer review

Not commissioned, externally peer-reviewed.

## Declaration of competing interest

None declared.
